# Detection of *Salmonella* Mbandaka Carrying the *bla*_CTX-M-8_ Gene Located on IncI1 Plasmid Isolated from a Broiler Flock Environment

**DOI:** 10.3390/pathogens13090723

**Published:** 2024-08-27

**Authors:** Magdalena Zając, Magdalena Skarżyńska, Anna Lalak, Ewelina Iwan, Dariusz Wasyl

**Affiliations:** 1 Department of Microbiology, National Veterinary Research Institute, 24-100 Pulawy, Poland; magdalena.skarzynska@piwet.pulawy.pl (M.S.); anna.lalak@piwet.pulawy.pl (A.L.); wasyl@piwet.pulawy.pl (D.W.); 2 Department of Omics Analyses, National Veterinary Research Institute, 24-100 Pulawy, Poland; ewelina.iwan@piwet.pulawy.pl

**Keywords:** *Salmonella*, ESBL, WGS, antimicrobial resistance, poultry

## Abstract

*Salmonella* Mbandaka is one of the most globally widespread serovars, occurring in many sources and included among twenty serovars that contribute to human salmonellosis in Europe. In Poland, it has been noted in non-human sources since 1996, being found firstly in feeds and later in waterfowl and chicken. Over the years, it gained epidemiological importance, being isolated from a wide range of animal species, including livestock. Generally, it is characterized by sensitivity to most antimicrobials and the ability to form biofilms. The occurrence of cephalosporin-resistant *Salmonella* in non-human sources is an extremely rare phenomenon in Poland. In this report, we characterized the full genome of the ESBL-producing *S*. Mbandaka strain isolated from a broiler farm environment (boot swab sample) in Poland in 2022. The isolate was serotyped as *S*. Mbandaka according to the White–Kaufmann–Le Minor scheme. Antimicrobial susceptibility testing performed with the microbroth dilution method showed its resistance to ampicillin, cefotaxime, ceftazidime, ciprofloxacin, and nalidixic acid. The whole-genome sequence was reconstructed using short and long reads and assembled into the complete chromosome and three plasmids: IncI1 pST113 (89,439 bp), Col(pHAD28) (2699 bp), and Col440 (2495 bp). The strain belonged to sequence type ST413. Plasmid analysis showed *bla*_CTX-M-8_ mobilization on IncI1(alpha) surrounded with insertion sequences. The analyzed genome content draws attention to the possibility of the horizontal spread of the resistance genes. To the best of our knowledge, this is the first report of *bla*_CTX-M-8_-positive *Salmonella* in Poland.

## 1. Introduction

Bacteria belonging to the *Salmonella* (*S*.) genus are still the subject of many studies, including monitoring and control programs, outbreak investigations, and genomic studies. In Europe, it was estimated that in 2022, nearly 65,208 gastroenteric infections were caused by *Salmonella* in humans, with *S*. Enteritidis playing the most important role [1]. 

*S*. Mbandaka is one of the most globally widespread serovars, found in many sources, and one of the top twenty serovars responsible for human salmonellosis in European countries in 2022 [1]. Generally, it is characterized by a high sensitivity to many antimicrobials, the ability to produce a biofilm, and adaptation to the proficient utilization of metabolites found in soya beans [2]. In Poland, it was first identified in 1996, and it has been isolated from non-human sources comprising a wide range of animal species, including livestock (waterfowl and hens) and feed [3,4]. Since the first reports, it has gained epidemiological importance and has been responsible for several human infections [5]. The main source of *S*. Mbandaka in Poland remains feed, followed by poultry, whereas, in the United Kingdom and France, this serovar is more associated with cattle, and feed (UK) or poultry (France) [2,6]. In recent years, outbreaks associated with this serovar have been reported. In 2018, a *S*. Mbandaka outbreak was linked to contaminated ready-to-eat (RTE) breakfast cereal products in the United States [7]. Between 2021 and 2023, *S*. Mbandaka caused a multicountry outbreak in Europe, which was related to chicken products [8].

Increasing antimicrobial resistance in pathogenic bacteria, including *Salmonella*, has become a serious global issue and causes treatment failures in human infections. Attention has been drawn to resistance to medically important antimicrobials (MIAs), including 3rd- and 4th-generation cephalosporins [9]. One of the most common mechanisms of resistance to cephalosporins is the production of extended-spectrum beta-lactamases (ESBLs). In 1999, the first case of ESBL-producing *S*. Mbandaka carrying the *bla*_CTX-M-3_ gene was documented in humans in Poland [10]. Meanwhile, the *bla*_CTX-M-8_ gene, which has been identified in various enterobacterial species worldwide [11,12,13,14], had not been previously detected in *Salmonella* from animal sources in Poland. This report shows the comprehensive genome characterization of the CTX-M-8-producing *S*. Mbandaka strain isolated from a broiler flock, marking the first occurrence of such a finding in Poland.

## 2. Materials and Methods

### 2.1. Bacterial Isolate, Serotyping, and Antimicrobial Testing

The strain S22_2161 was isolated in 2022 from a broiler flock boot swab sample and tested according to PN-EN ISO 6579-1:2017-04/A1:2020 [15] as part of the National *Salmonella* Control Program. One of the official laboratories referred the isolate to the National Reference Laboratory for Salmonellosis and Antimicrobial Resistance in the National Veterinary Research Institute for confirmation and antimicrobial resistance (AMR) testing, as described in the Commission Implementing Decisions 2020/1729 (https://eur-lex.europa.eu/eli/dec_impl/2020/1729/oj). The pure culture was confirmed to genus level using matrix-assisted laser desorption ionization time-of-flight mass spectrometry (MALDI-TOF, Bruker Daltonics GmbH, Bremen, Germany) using the extraction method, following the producer guidelines (Bruker Daltonics GmbH, Bremen, Germany), and serotyped according to the White–Kaufmann–Le Minor scheme [16]. Antimicrobial resistance testing was performed via the microbroth dilution method (Sensititre EUVSEC plates; TREK Diagnostic Systems, Thermo Fisher Scientific, Waltham, MA, USA) with two antimicrobial panels (EUVSEC3 and EUVSEC2) used in the official antimicrobial resistance monitoring scheme in the EU (Tables 2 and 5 of the Annex to (UE) 2020/1729). Epidemiological cut-off values (ECOFFs) for minimal inhibitory concentration (MICs) listed in the legislation as mentioned above were applied for compounds representing nine antimicrobial classes: aminoglycosides (amikacin, gentamicin), beta-lactams (ampicillin, cefotaxime, ceftazidime, imipenem, ertapenem, meropenem), folate-path inhibitors (trimethoprim, sulfamethoxazole), glycylcyclines (tigecycline), macrolides (azithromycin), phenicols (chloramphenicol), polymyxins (colistin), tetracyclines (tetracycline), and quinolones (ciprofloxacin, nalidixic acid). *E. coli* ATCC 25922 was used as the reference strain in MIC analyses in parallel with the tested isolate. MIC values above the cut-off were described as resistant (non-wild-type, NWT).

### 2.2. Whole-Genome Sequencing and Bioinformatical Analysis

DNA was extracted from overnight pure nutrient agar culture at 37 °C using the Maxwell Rapid Sample Concentrator (RSC) cultured cell DNA Kit (Promega, Madison, WI, USA). The quantity and quality of DNA were assessed using the Qubit 3.0 (Thermo Fisher Scientific, Waltham, MA, USA) and capillary electrophoresis using a Fragment Analyzer (Agilent, Santa Clara, CA, USA). Short- and long-fragment libraries were constructed using the KAPA HyperPlus Kit (Roche, Basel, Switzerland) and Ligation sequencing kits (SQK-LSK109; Oxford Nanopore, Oxford, UK), respectively. Whole-genome sequencing was performed on the MiSeq (v3 2 × 300 bp, Illumina, San Diego, CA, USA) and MinION (Oxford Nanopore) in parallel. The trimming of short reads was performed by using fastp 0.20.0 (https://github.com/OpenGene/fastp) [17], and the trimming of long reads was conducted with Porechop 0.2.4 (https://github.com/rrwick/Porechop). The genome was assembled by Unicycler v0.4.8 (https://github.com/rrwick/Unicycler). Bioinformatic tools from the Center of Genomic Epidemiology (CGE) (http://www.genomicepidemiology.org/services, accessed 7 December 2023) were used to determine the MLST type (MLST 2.0) [18], the plasmid replicons (PlasmidFinder 2.1; thresholds: 95% identity and 80% coverage), the plasmid MLST (pMLST 2.0; database version: 24 April 2023) [19], the resistance genes, and the mobile genetic elements (MGE v1.0.3; database version: v1.0.2; 95% identity and 90% coverage) [20]. Proksee software (https://proksee.ca/, accessed 20 May 2024) [21] was used for the visualization of the genes, BLAST comparisons (BLAST Formatter 1.0.3), ANI calculations (FastANI 1.1.0) [22], resistance gene identification with CARD 1.2.1 [23], and gene annotations with Prokka 1.1.1 [24] and Bakta 1.0.0 [25]. Core-genome MLST (cgMLST) and HierCC [26] were performed using the *S. enterica* cgMLST scheme of 3002 target loci available on the Enterobase (https://enterobase.warwick.ac.uk/, accessed on 19 February 2024). The comparison was performed on 19 February 2024 using the available 1176 genomic sequences of *Salmonella* Mbandaka deposited in the Enterobase (https://enterobase.warwick.ac.uk/). We searched for isolates with “Europe” listed as a continent of isolate origin in the strain metadata, “Mbandaka” as the serovar in either the strain metadata or experimental data, and “ST413” in the experimental data. The available complete sequences of IncI1 plasmids carrying the *bla*_CTX-M-8_ gene were downloaded from the National Center for Biotechnology Information (NCBI).

## 3. Results

The isolate was confirmed as *Salmonella enterica* subsp. *enterica* serovar Mbandaka. Antimicrobial susceptibility testing revealed its resistance to ampicillin (>32 mg/L), cefotaxime (32 mg/L), ceftazidime (4 mg/L), ciprofloxacin (0.5 mg/L), and nalidixic acid (16 mg/L). The isolate was susceptible to amikacin (≤4 mg/L), cefoxitin (4 mg/L), chloramphenicol (≤8 mg/L), gentamicin (≤0.5 mg/L), imipenem (≤0.12 mg/L), meropenem (≤0.03 mg/L), tetracycline (≤2 mg/L), and trimethoprim (≤0.25 mg/L). The MIC values for antimicrobials or a combination of substances with a lack of interpretation criteria were as follows: azithromycin (4 mg/L), cefepime (16 mg/L), cefotaxime-clavulanate (0.12 mg/L), ceftazidime-clavulanate (0.5 mg/L), colistin (≤1 mg/L), ertapenem (≤0.015 mg/L), sulfamethoxazole (16 mg/L), temocillin (8 mg/L), and tigecycline (≤0.25 mg/L).

**Figure 1:**
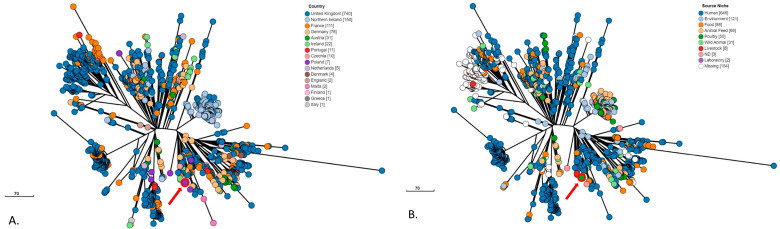
A minimum spanning tree based on cgMLST and HierCC analysis of 1176 *S*. Mbandaka ST413 strains identified in Europe between 1993 and 2023. The tree was generated using the Enterobase (https://enterobase.warwick.ac.uk/, accessed 19 February 2024). Isolates are colored by (**A**) country of origin, and (**B**) source, as shown in the legend. The tested isolate S22_2161 is marked with a red circle and arrow.

The genome (4,845,043 bp) was assembled into the complete chromosome and three plasmids: IncI1(alpha) (89,439 bp), Col(pHAD28) (2699 bp), and Col440 (2495 bp). The strain belonged to sequence type ST413. The phylogenetic analysis of 1176 *S*. Mbandaka ST413 genomes derived from 16 European countries and isolated between 1993 and 2023 showed that isolate S22_2161 clustered with strains mainly from the United Kingdom, then from Germany, Ireland, Poland, and Czechia (Figure 1A). These isolates were derived from a variety of sources, with human ones being the most numerous (Figure 1B).

**Figure 2:**
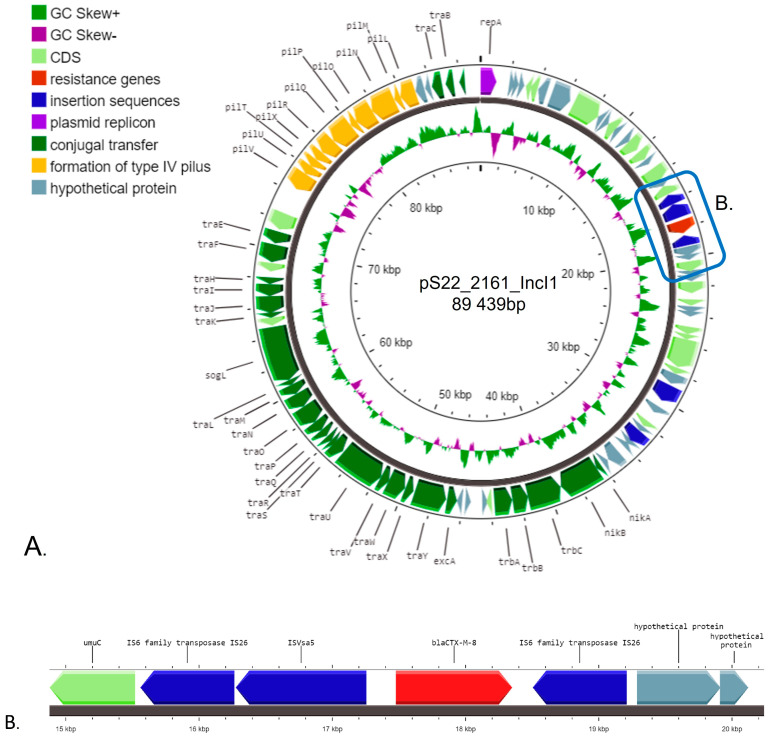
Diagrams of pS22_2161_IncI1 structure: (**A**) genetic map of analyzed plasmid, and (**B**) the genetic environment of the *bla*_CTX-M-8_ gene. The genes classified into the same group are indicated by the same colors.

The IncI1(alpha) plasmid was identified as pST113 and found to carry the *bla*_CTX-M-8_ gene in a module consisting of IS26-ISVsa5(=IS10R)-*bla*_CTX-M-8_-IS26, associated with phenotypic resistance to ampicillin, cefotaxime, ceftazidime, and cefepime (Figure 2A,B). It was the only resistance gene found on this plasmid. The module was located between *umuC* and hypothetical protein genes. The conjugal transfer system genes containing *trb*/*tra* and *pil* gene clusters were also identified (Figure 2A).

**Figure 3:**
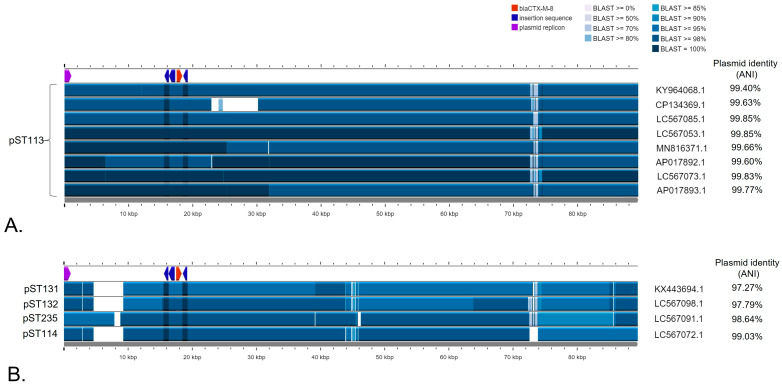
Comparison of the pS22/2161_IncI1 plasmid with (**A**) eight IncI1 plasmids belonging to pST113 harboring *bla*_CTX-M-8_, and (**B**) four plasmids belonging to pST114, pST131, pST132, and pST235 carrying the *bla*_CTX-M-8_ gene. pS22_2161_IncI1 was used as a reference. Color shades correspond to the existing percent of identity with the reference genome. This figure was generated by the Proksee tool with BLASTn.

Comparative analysis of the studied IncI1 plasmid with other pST113 IncI1 sequences (Table 1) indicated that *bla*_CTX-M-8_ was inserted in an identical order in all plasmids of *E. coli* strains isolated from different sources, varying from chicken meat to humans, and derived from geographically distant continents and countries, like Portugal, Japan, and Brazil (Figure 3A). A comparison of the tested plasmid with the ones carrying *bla*_CTX-M-8_ but belonging to other plasmid sequence types (Table 1) also revealed the occurrence of the same gene module consisting of *bla*_CTX-M-8_ (Figure 3B). The average nucleotide identity (ANI) of the pST113 plasmids ranged between 99.4% (KY964068.1) and 99.87% (LC567053.1) (Figure 3A), whereas the comparison of other pST plasmids with the studied pS22_2161_IncI1 revealed variation of between 97.27% (KX443694.1) and 99.03% (LC567072.1) (Figure 3B).

The Col(pHAD28) plasmid harbored the *qnrB19* gene conferring ciprofloxacin resistance. Moreover, amino acid substitution T57S in ParC was identified on the chromosome and might explain the elevated ciprofloxacin MIC value of the isolate. The smallest plasmid Col440 did not carry any resistance genes.

## 4. Discussion

The occurrence of cephalosporin-resistant *Salmonella* in non-human sources is an extremely rare phenomenon in Poland. To the best of our knowledge, the only described case was CTX-M-25-producing, multidrug-resistant *S*. Kentucky, another unique bacterium–gene–plasmid combination found in our previous study [31]. In Europe, the overall *Salmonella* resistance to third-generation cephalosporins is noted at very low levels both in humans and food-producing animals [29]. It is worth noting that higher resistance levels were reported in isolates from humans, and differences in specific *Salmonella* serovars were observed: *S.* Kentucky (12.3%), *S.* Infantis (6.0%), or monophasic *S*. Typhimurium (1.8%) [32]. In Poland, *Salmonella* ESBL producers isolated from humans belonged mostly to *S*. Enteritidis, *S*. Thompson, and *S*. Typhimurium expressing mostly CTX-M-3 or SHV-5 [33]. The currently reported strain revealed combined resistance to ciprofloxacin, cefotaxime, and ceftazidime resulting from the presence of genes transmitted on mobile genetic elements. Since the strain was revealed within official antimicrobial resistance monitoring, its detection was mentioned in the EFSA report [32]. It should be emphasized that, similar to cephalosporins, fluoroquinolones also belong to the category of medically important antimicrobials. Simultaneous resistance to both MIA classes may increase the risk of unsuccessful treatment for potential infection caused by the specific *S*. Mbandaka strain.

Our literature search drew attention to the cosmopolitan character of CTX-M-8 extended-spectrum beta-lactamase. Resistance to cephalosporins resulting from the presence of *bla*_CTX-M-8_ was reported for the first time in Brazil, where it was identified in three different clinical isolates of *Enterobacter aerogenes*, *Enterobacter cloacae*, and *Citrobacter amalonaticus* [34]. In Japan, CTX-M-8-producing *E. coli* has been mainly found in retail chicken meat imported from Brazil and patients in clinical settings, as well as being isolated from livestock [12]. In Europe, the gene was identified in different bacteria, mainly *E. coli*, but also in *Salmonella* spp. and *Enterobacter aerogenes* [11,32,35].

It should be remembered that the focus should be placed not only on the bacterial species but, perhaps even more importantly, on the dissemination route of the *bla*_CTX-M_ genes and carrier plasmids. Considering the transmission of the *bla*_CTX-M_ family, IncI1 is one of the most prevalent ESBL-related plasmids in Europe [36]. It has been found worldwide in Enterobacterales isolated from a wide variety of sources, including humans. It has also been confirmed that the plasmid carries a range of different resistance genes [36]. Among different plasmid sequence types, pST113 appears to be the most associated with the *bla*_CTX-M-8_ gene transmission [11,12]. The presence of an identical set of the genes surrounding *bla*_CTX-M-8_ in other pST plasmids, i.e., pST114, pST115, or pST235, confirms the possibility of its dissemination by less-known IncI1 plasmid types [11,12]. This observation also suggests high mobility due to the presence of insertion sequence IS26, belonging to the IS26 family, which plays a crucial role in the transmission of antibiotic resistance determinants in Gram-negative bacteria [37]. It is also the best-characterized insertion sequence in the IS26 family, which includes, e.g., IS1216, IS1006, and IS1008, which are involved in the transmission of resistance genes in bacterial pathogens [37]. The presence of *bla*_CTX-M-8_-IncI1 derived mainly from *E. coli* may suggest that tested *Salmonella* could acquire this resistance from an *E. coli* strain. To date, research conducted on *E. coli* in food-producing animals and wildlife in Poland has proven the occurrence of various CTX-M variants but not CTX-M-8 [38,39,40,41].

The finding of a rare CTX-M gene in the tested strain draws attention to plasmids that can be easily acquired and spread. In this case, it can be an important factor influencing the genetic adaptation of *S*. Mbandaka by increasing its resistance. The tested isolate belonged to a common sequence type disseminated among European countries in many sources, including humans. The clustering together mainly with UK isolates could be due to the best availability of genomes from this country. The analyzed genome content of the studied isolate showed the problem of a potential increase in epidemiological importance of this serovar due to acquired resistance genes. The incidence of *Salmonella* carrying cephalosporin resistance genes located on mobile genetic elements (MGEs) represents a significant threat to public health in a One Health context.

## Figures and Tables

**Table 1:** IncI1 plasmids carrying the *bla*_CTX-M-8_ gene obtained from the NCBI database and used for comparison in this study.

No.	Plasmid	Species	Source	Geographic Location	Collection Year	Plasmid Size (bp)	pMLST	Accession No.	Reference
1	pS22_2161_IncI1	*Salmonella* Mbandaka	Broiler flock environment	Poland	2022	89,439	pST113	CP146619.1	This study
2	pLV23529-CTX-M-8	*Escherichia coli*	*Sus scrofa*	Portugal	2015	89,458	pST113	KY964068.1	[27]
3	pA117-CTX-M-8	*Escherichia coli*	*Loxodonta africana*	Brazil	2019	101,273	pST113	MN816371.1	[28]
4	pN23	*Escherichia coli*	*Homo sapiens*	Japan	No data	91,831	pST113	AP017892.1	[12]
5	pS11	*Escherichia coli*	Chicken meat	Japan	No data	101,377	pST113	AP017893.1	[12]
6	pMTY12368_IncI1-I	*Escherichia coli*	Chicken meat	Japan	2012	84,532	pST113	CP134369.1	unpublished
7	pHU493	*Escherichia coli*	*Homo sapiens*	Japan	2010	94 912	pST113	LC567073.1	[29]
8	pCH41	*Escherichia coli*	Chicken meat	Japan	2010	86,204	pST113	LC567085.1	[29]
9	pP44	*Escherichia coli*	*Canis lupus familiaris*	Japan	2015	89,476	pST113	LC567053.1	[29]
10	pHU485	*Escherichia coli*	*Homo sapiens*	Japan	2010	86,204	pST114	LC567072.1	[29]
11	pCH110	*Escherichia coli*	Chicken meat	Japan	2010	97,607	pST235	LC567091.1	[29]
12	pCH365	*Escherichia coli*	Chicken meat	Japan	2010	108,776	pST132	LC567098.1	[29]
13	pICBEC72Hctx	*Escherichia coli*	*Homo sapiens*	Brazil	2016	92,070	pST131	KX443694.1	[30]

## Data Availability

The original contributions presented in the study are included in the article, further inquiries can be directed to the corresponding author. The complete genome of Salmonella Mbandaka strain S22_2161 was assigned the GenBank project number PRJNA1082544 (CP146618.1–CP146621.1).
